# Circulating Levels of Endothelin-1 and Big Endothelin-1 in Patients with Essential Hypertension

**DOI:** 10.3390/pathophysiology28040031

**Published:** 2021-10-25

**Authors:** Krasimir Kostov, Alexander Blazhev

**Affiliations:** 1 Department of Pathophysiology, Medical University-Pleven, 1 Kliment Ohridski Str., 5800 Pleven, Bulgaria; 2 Department of Biology, Medical University-Pleven, 1 Kliment Ohridski Str., 5800 Pleven, Bulgaria; yalishanda9@gmail.com

**Keywords:** essential hypertension, endothelin-1, big endothelin-1

## Abstract

The role of endothelin-1 (ET-1) in the pathogenesis of hypertension (HTN) is not clearly established. There is evidence that its circulating levels are elevated in some forms of experimental and human HTN, but this was not a consistent finding. Based on these controversial data, we tested serum levels of ET-1 and Big ET-1 (the precursor of ET-1) in patients with essential HTN, comparing the results with those of healthy normotensive controls. The levels of ET-1 and Big ET-1 were measured by ELISA. Our results in patients with essential HTN showed that the mean levels of ET-1 (5.01 ± 2.1 pg/mL) were significantly higher (*F* = 6.34, *p* = 0.0144) than the mean levels in the control group (3.2 ± 1.0 pg/mL). The levels of Big ET-1 in patients with essential HTN (0.377 ± 0.1 pmol/L) were similar to those in the control group (0.378 ± 0.07 pmol/L) and did not differ significantly (*F* = 0.00, *p* = 0.9531). These data suggest that ET-1, but not Big ET-1, may play an important role in the pathogenesis of primary HTN.

## 1. Introduction

Hypertension (HTN) is one of the most prevalent diseases worldwide and is among the most important risk factors for cardiovascular and cerebrovascular complications [1]. It is currently thought to be the result of disturbances in a number of neural, renal, hormonal, and vascular mechanisms regulating blood pressure (BP) [2], as crucial importance is given to the imbalance of a number of vasoactive substances [3]. In HTN, the delicate balance in the regulation of vascular tone is disturbed due to decreased bioavailability of NO and the overproduction of ET-1 [4,5].

ET-1 is a vasoactive peptide identified in 1988 by Yanagisawa and colleagues from the supernatant of porcine aortic endothelial cells (ECs). It is composed of 21 amino acids and two intrachain disulfide linkages in the molecule [6]. In the vasculature, ET-1 acts on ETA and ETB (ETB1 and ETB2) receptors located on the vascular smooth muscle cells (VSMCs) and ECs to induce vascular contraction or vasodilation [7]. Vasoconstrictive action of ET-1 is mainly mediated through ETA on VSMCs, while vasodilation is mediated through ETB1 on ECs [8].

The role of ET-1 in the pathogenesis of HTN is not clearly established. It is assumed that under physiological conditions, the vasodilating action of ET-1 may predominate, whereas under pathophysiological conditions, ET-1 may behave as a vasoconstrictor and play a role in the pathophysiology of HTN [9]. There is evidence that circulating levels of ET-1 are elevated in some forms of experimental and human HTN, but this was not a consistent finding in all forms of HTN [10]. Furthermore, some studies show that Big ET-1, the biological precursor of ET-1, may be a more accurate indicator of the degree of activation of the endothelin system compared to ET-1, as it has a longer half-life and slower clearance than ET-1 [11]. However, there are currently insufficient studies on Big ET-1 levels in patients with essential HTN. Based on these controversial data, we tested serum concentrations of ET-1 and Big ET-1 in patients with essential HTN, comparing the results with those of healthy normotensive controls.

## 2. Patients and Methods

### 2.1. Characteristics of the Study Population

The study population consisted of 80 persons: normotensive control group (*n* = 20; mean age 47.9 ± 11.3 years) and hypertensive group, patients who were treated at the University Hospital “Dr. Georgi Stranski”—Pleven, Bulgaria (*n* = 60; mean age 65.3 ± 11.5 years). The serum samples of patients and controls were taken from 17 to 21 February 2015. The clinical characteristics of the groups are shown in Table 1.

### 2.2. Inclusion and Exclusion Criteria in the Study

#### 2.2.1. Inclusion Criteria for Patients

- Male or female aged ≥35 years and ≤80 years;
- Patients with essential HTN (either SBP ≥ 140 mmHg, DBP ≥ 90 mmHg or both);
- Willingness to voluntarily participate in the study and sign an informed consent form.

#### 2.2.2. Exclusion Criteria for Patients

- Male or female aged ≤35 years and ≥80 years;
- Secondary HTN and pulmonary HTN;
- Chronic renal disease, heart failure, liver dysfunction, and malignant tumor;
- Inability or unwillingness to participate in the study or to sign an informed consent form.

#### 2.2.3. Inclusion Criteria for Control Subjects

- Healthy men or women aged ≥35 years and ≤80 years;
- Individuals with normal BP (SBP 120–129 mmHg and DBP 80–84 mmHg);
- Willingness to voluntarily participate in the study and sign an informed consent form.

### 2.3. Blood Pressure Measurement

BP was measured using a standard cuff mercury sphygmomanometer on the left arm in a sitting position, after 5–10 min rest. The assessment of the arterial HTN was made according to the 2018 ESC/ESH Guidelines for the management of arterial HTN [12]. Normal BP was defined as SBP 120–129 mmHg and DBP 80–84 mmHg. HTN was defined as either SBP ≥ 140 mmHg, DBP ≥ 90 mmHg or both.

### 2.4. Immunological Assays

To measure the concentrations of ET-1 and Big ET-1, blood was drawn into serum tubes and was centrifuged at 2500 rpm for 10 min. Until immunological assay was performed, the serums were stored at −70 °C. The concentrations were measured by ELISA method using commercially available kits.

#### 2.4.1. Determination of ET-1

To measure ET-1 concentrations, an ELISA kit from Biomedica Medizinprodukte GmbH & Co. KG, Vienna, Austria (Cat. No. BI-20052) was used according to the manufacturer’s instructions. Serum samples were assayed at 450 nm on an automatic micro-ELISA plate reader (Coulter Microplate Reader UV Max, Molecular Devices Corp., Menlo Park, CA, USA).

#### 2.4.2. Determination of Big ET-1

To measure Big ET-1 concentrations, an ELISA kit from Biomedica Medizinprodukte GmbH & Co. KG, Vienna, Austria (Cat. No. BI-20082H) was used according to the manufacturer’s instructions. Serum samples were assayed at 450 nm on an automatic micro-ELISA plate reader (Coulter Microplate Reader UV Max, Molecular Devices Corp., Menlo Park, CA, USA).

### 2.5. Statistical Analysis

Statistical analyses were performed using Statgraphics Centurion XVI software (Statpoint Technologies, Inc., Warrenton, VA, USA). The data were expressed as mean ± standard deviation (SD). The differences between the groups were assessed by Fisher’s *F*-test (ANOVA). Values of *p* < 0.05 were considered statistically significant.

## 3. Results

### 3.1. Comparison of Serum Levels of ET-1 between the Hypertensive Group and the Control Group

**Figure 1:**
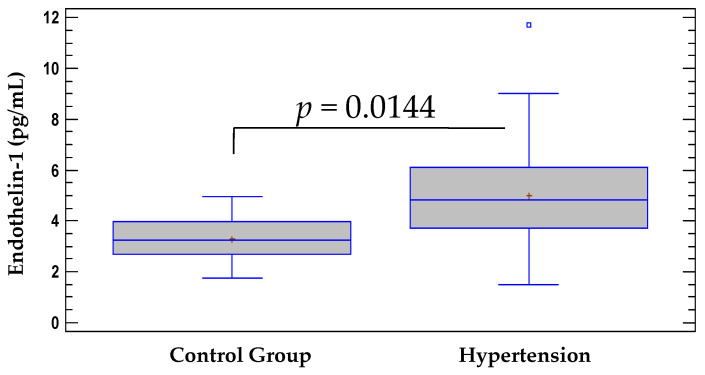
Serum levels of ET-1 in hypertensive group vs. control group. Data are represented as mean ± SD.

Studies in healthy adults have shown that basal plasma and serum ET-1 levels are normally between 0.7 and 5 pg/mL [8,13]. The results of our study in patients with essential HTN revealed that the mean serum levels of ET-1 (5.01 ± 2.1 pg/mL) were significantly higher (*F* = 6.34, *p* = 0.0144) than the mean levels in the control group (3.2 ± 1.0 pg/mL) (Figure 1). These data suggest that elevated levels of ET-1 could play an important role in the development of primary HTN.

### 3.2. Comparison of Serum Levels of Big ET-1 between the Hypertensive Group and the Control Group

**Figure 2:**
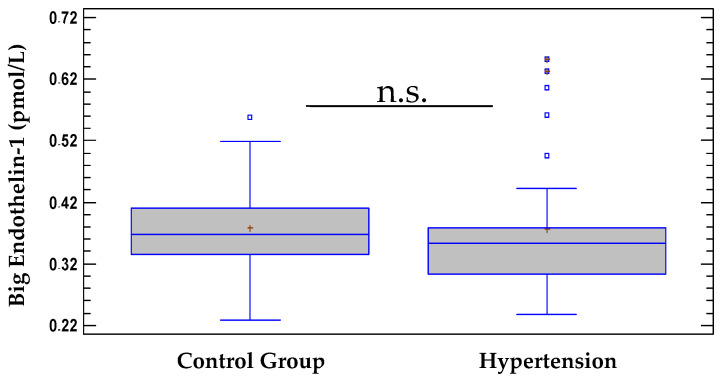
Serum levels of Big ET-1 in hypertensive group vs. control group. Data are represented as mean ± SD. n.s.—not significant (*p* > 0.05).

The levels of Big ET-1 in patients with essential HTN (0.377 ± 0.1 pmol/L) were similar to those in the control group (0.378 ± 0.07 pmol/L) and did not differ significantly (*F* = 0.00, *p* = 0.9531) (Figure 2). These data suggest that Big ET-1 is unlikely to be causally linked to the development of primary HTN.

According to the results discussed above, probably ET-1, but not Big ET-1, may play an important role in the development of essential HTN. The controversial and not always consistent results regarding elevated ET-1 levels in patients with primary HTN are probably related to two main reasons. The first is that its elimination from the blood is too fast (plasma half-life 1–2 min) [14]. The second is that the secretion of ET-1 by ECs is polarized mainly to the underlying VSMCs, leading to a minimal increase in its circulating levels [8]. Other possible causes for these disparate results are the specificity of the antibodies used in the immunoassay, the degree of cardiovascular damage, dietary salt intake, obesity, diabetes, and race [15].

## 4. Discussion

One of the first comparisons of ET-1 concentrations in people with HTN was made between pheochromocytoma patients and healthy controls. Higher levels of ET-1 were observed in patients with pheochromocytoma. In this report, the authors note that HTN in patients with pheochromocytoma is mainly catecholamine-dependent, but may be secondarily ET-1-dependent [16]. These data are supported by previous reported cases in patients with hemangioendothelioma who have significantly elevated ET-1 levels along with HTN [17]. Elevated ET-1 levels and high BP in patients from these studies returned to normal after surgical removal of the tumors [16,17]. Furthermore, resistant HTN with elevated ET-1 levels has been observed more frequently in patients of African-American descent or those with obesity, in whom the risk of developing cardiovascular and renal diseases is increased [18]. Furthermore, in individuals with normal BP, high plasma ET-1 levels are associated with the development of HTN [19]. The role of ET-1 in the development of the hypertensive process is also supported by data in patients with essential HTN or resistant HTN, which show that when treated with a non-selective ET-receptor antagonist bosentan [20] or with the selective ETA receptor antagonist darusentan [21,22,23], BP is significantly reduced.

The results of our study in patients with essential HTN showed significantly higher serum concentrations of ET-1 compared to normotensive controls (Figure 1). Elevated ET-1 levels in patients with HTN are consistent with some previous clinical studies [24,25,26,27,28,29], as well as with our previous results, which showed that serum ET-1 concentrations were significantly elevated in patients with high BP [10,30]. Other authors have reported that the levels of ET-1 are normal in patients with essential HTN, but point out that the local levels of ET-1 in the vascular wall are elevated [18,31,32]. This may be due to the specific mechanisms of action of different groups of antihypertensive drugs, which is associated with different effects on circulating ET-1 levels. For example, moxonidine therapy did not affect ET-1 levels, while losartan significantly reduced levels [33].

In contrast to the concentrations of ET-1, which were significantly higher in patients with HTN, the concentrations of the precursor Big ET-1 did not differ significantly from those of controls (Figure 2). Probably, elevated levels of Big ET-1 in patients with HTN can be detected when there is either concomitant heart failure [34,35], pulmonary HTN [36,37], coronary artery disease [38,39], or in combination. Interestingly, Big ET-1 and ET-1 levels were high in patients with arsenic (As)-induced HTN, which is thought to be mediated by oxidative stress as a result of chronic As exposure [11].

In addition to changes in ET-1 levels, endothelial dysfunction at the onset of HTN has also been documented through the use of other circulating biomarkers, such as asymmetric dimethylarginine (ADMA), oxidized LDL, and endothelial microvesicles. Over the last years, ADMA, the endogenous inhibitor of endothelial nitric oxide synthase (NOS3), has emerged as a cardiovascular molecule reflecting endothelial dysfunction and the vascular changes observed in essential HTN [40,41].

## 5. Conclusions

In summary, our results indicate that serum ET-1 levels are elevated in patients with essential HTN and could play an important role in the development of the hypertensive process. Increased production of ET-1 in the vascular wall may promote oxidative stress and low-grade inflammation, with the development of endothelial dysfunction and increased vasoconstrictor activity. Increased ET-1 production can contribute to arterial aging and the development of atherosclerotic changes, which are associated with increased arterial stiffness and manifestation of isolated systolic HTN. In addition, ET-1 is involved in the complex regulation of BP through synergistic interactions with angiotensin II, regulates the production of catecholamines and sympathetic activity, affects renal hemodynamics and water–salt balance, and regulates baroreceptor activity and myocardial contractility. All these effects of ET-1 in the conditions of its increased production can lead to a permanent increase in BP and the development of HTN [42]. The levels of Big ET-1 in hypertensive patients were similar to those in the control group, suggesting that Big ET-1 is unlikely to be causally related to the development of primary HTN. Taken together, our results indicate that activation of the endothelin system may be an important cause for the clinical manifestation of essential HTN.

## Figures and Tables

**Table 1:** Clinical characteristics of the groups.

Number of Examined Individuals (*n* = 80)	Control Group (*n* = 20)	Hypertensive Group (*n* = 60)
Sex, Male/Female	10/10	24/36
Age, years ^1^	47.9 ± 11.3	65.3 ± 11.5
Duration of HTN ^1^	N/A ^2^	8.6 ± 5.9
SBP, mmHg ^1^	124.0 ± 3.7	155.4 ± 4.8
DBP, mmHg ^1^	82.2 ± 4.1	87.1 ± 2.6
ET-1, pg/mL ^1^	3.2 ± 1.0	5.01 ± 2.1
Big ET-1, pmol/L ^1^	0.378 ± 0.07	0.377 ± 0.1
TC, mmol/L ^1^	3.9 ± 0.7	4.8 ± 1.2
LDL-C, mmol/L ^1^	2.5 ± 0.6	3.2 ± 1.1
HDL-C, mmol/L ^1^	1.1 ± 0.3	1.02 ± 0.2
TG, mmol/L ^1^	1.3 ± 0.6	1.5 ± 1.3
CRP, mg/L ^1^	1.07 ± 0.9	7.5 ± 9.6
Hypertensive CVD:	N/A ^2^	(*n* = 12)
-Coronary Artery Disease	N/A ^2^	(*n* = 5)
-Kidney Damage	N/A ^2^	(*n* = 3)
-Brain Damage	N/A ^2^	(*n* = 2)
-Eye Damage	N/A ^2^	(*n* = 2)

^1^ Mean ± SD; ^2^ N/A, not available; SBP, systolic blood pressure; DBP, diastolic blood pressure; ET-1, endothelin-1; Big ET-1, Big endothelin-1; TC, total cholesterol; LDL-C, low-density lipoprotein cholesterol; HDL-C, high-density lipoprotein cholesterol; TG, triglyceride; CRP, C-reactive protein; CVD, cardiovascular disease.

## Data Availability

The authors confirm that the data supporting the findings of this report are available within the article.
